# Hydrangenol suppresses VEGF-stimulated angiogenesis by targeting p27KIP1-dependent G1-cell cycle arrest, VEGFR-2-mediated signaling, and MMP-2 expression^*^

**DOI:** 10.1080/19768354.2019.1578262

**Published:** 2019-02-14

**Authors:** Yujeong Gho, Seung-Shick Shin, Yung Hyun Choi, Kisung Ko, Wun-Jae Kim, Sung-Kwon Moon

**Affiliations:** a Department of Food and Nutrition, Chung-Ang University, Anseong, Republic of Korea; b Department of Food Science and Nutrition, Jeju National University, Jeju, Republic of Korea; c Department of Biochemistry, College of Oriental Medicine, Dongeui University, Busan, Republic of Korea; d Department of Medicine, College of Medicine, Chung-Ang University, Seoul, Republic of Korea; e Department of Urology, Chungbuk National University, Cheongju, Republic of Korea

**Keywords:** Hydrangenol, angiogenesis, HUVECs, VEGF, *ex vivo* aortic ring

## Abstract

We previously reported that hydrangenol has potent antitumor activity against human bladder cancer EJ cells. Here, we investigated the antiangiogenic activity of hydrangenol using *in vitro* and *ex vivo* models. Treatment with hydrangenol significantly inhibited the proliferation of vascular endothelial growth factor (VEGF)-induced HUVECs in a concentration-dependent manner (EC_50 _= 10 μM). Flow cytometry analysis revealed that hydrangenol suppressed the VEGF-induced inhibition of G1-cell cycle phase and also decreased cyclin D1, cyclin E, CDK2, and CDK4 levels. Hydrangenol-mediated arrest in the G1-cell cycle phase was associated with p27KIP1 level, but not p21WAF1 or p53 level. Hydrangenol also significantly inhibited VEGFR-2-mediated signaling pathways including ERK1/2, AKT, and endothelial nitric oxide synthase. Interestingly, immunoprecipitation assay demonstrated that the inhibition of VEGFR-2 activation was independent of VEGF binding, thereby suggesting an allosteric regulation of hydrangenol against VEGFR-2. Additionally, hydrangenol inhibited migration, invasion, and capillary-like tubular formation in VEGF-stimulated HUVECs. Zymography and immunoblot analyses revealed that these inhibitory activities were partially owing to the VEGF-induced inhibition of matrix metalloproteinase-2 activity. Finally, VEGF-mediated microvessel sprouting was inhibited in the presence of hydrangenol in *ex vivo* aortic ring assay. Taken together, hydrangenol possesses a potent antiangiogenesis potential; thus we believe that hydrangenol may be developed as a therapeutic reagent to treat angiogenesis-mediated diseases.

## Introduction

Angiogenesis or neovascularization is the process of new blood vessel formation from pre-existing endothelial cells. Its physiological role has been well characterized as a critical trigger for the neoplastic growth of tumors (Folkman 1971). The endothelium, which forms the inner lining of the blood vessels, plays a key role in the process of neovascularization, a multi-step process involving the proliferation, migration, and capillary-like tubular structure formation of endothelial cells (Yancopoulos et al. 2000). Under normal circumstance, angiogenesis is tightly controlled by a balance between pro- and antiangiogenic molecules (Bussolino et al. 1997). Vascular endothelial growth factor (VEGF), a well-characterized angiogenic stimulator, is the primary regulator of angiogenic processes (Yancopoulos et al. 2000). VEGF belongs to platelet-derived growth factor (PDGF) superfamily which is classified into five related growth factors: VEGF-A, VEGF-B, VEGF-C, VEGF-D, and placental growth factor (PGF) (Eichmann & Simons 2012) In response to VEGF, endothelial cells regulate angiogenesis by activating its receptors: VEGFR-1 (Flt-1) and VEGFR-2 (KDR/Flk-1 in mice). VEGFR-1 functions as a regulator of morphogenesis, whereas VEGFR-2 plays a role in mitogenesis, migration, and invasion of endothelial cells which is closely associated with angiogenic regulation. Upon binding of VEGF to VEGFR-2, endothelial cells trigger proliferative signaling pathways, which promote the degradation of basement membrane and extracellular matrix (ECM) by matrix metalloproteinase-2 (MMP-2), a key molecule controlling the migration and invasion of endothelial cells (Lamalice et al. 2007). In addition, VEGF activates early responsive intracellular signaling molecules including ERK1/2, AKT, and endothelial nitric oxide synthase (eNOS) in endothelial cells (Takahashi et al. 1999).

Hydrangenol, a naturally occurring dihydroisocoumarin, is mainly obtained from *Hydrangea macrophylla*. It has been utilized as a folk medicine in low doses as an oral antipyretic or antidiabetic agent (Liu et al. 2013). Recently, hydrangenol has gained popularity for its bioactivities including antimicrobial (Nozawa et al. 1981), antiallergic (Matsuda et al. 1999), antidiabetic (Zhang et al. 2007), antimalarial (Kamei et al. 2000), and anti-inflammatory (Kim et al. 2016) activities. Kim et al. have reported that hydrangenol exhibits a potent antiallergic activity by suppressing NF-κB-mediated inflammatory signaling in LPS-stimulated macrophages (Kim et al. 2016). In addition, we have previously reported that hydrangenol exhibited anticancer activity by inhibiting proliferation, migration, and invasion of bladder cancer EJ cells (Shin et al. 2018). Kawamori et al. have reported that hydrangenol significantly reduced the frequency of AOM-induced colonic aberrant crypt foci development in a rat model (Kawamori et al. 1995). Despite intensive studies on the bioactivity of hydrangenol, the mechanism of antiangiogenesis still remains elusive. Here, we have investigated the antiangiogenic activity of hydrangenol utilizing both *in vitro* and *ex vivo* systems. To our knowledge, this is the first study demonstrating the antiangiogenic activity of hydrangenol; thus we believe that our data may provide valuable information for the development of therapeutic reagents against angiogenesis-mediated diseases.

## Materials and methods

### Materials

Hydrangenol was purchased from CoreSciences Co. (#BBP01679, purity 98.5%, Seoul, Korea). Human recombinant VEGF was obtained from R&D Systems (Minneapolis, MN, USA). Antibodies against ERK1/2, AKT, eNOS, VEGFR-2, phospho-ERK1/2, phospho-AKT, phospho-eNOS, and phospho-VEGFR-2 were purchased from Cell Signaling Technology Inc. (Danvers, MA, USA). Polyclonal antibodies against cyclin D1, cyclin E, CDK2, CDK4, p21WAF1, p27KIP1, p53, and GAPDH were purchased from Santa Cruz Biotechnology Inc. (Santa Cruz, CA, USA). Polyclonal antibody against MMP-2 was purchased from Chemicon (Temecula, CA, USA).

### Cell culture

Human umbilical vein endothelial cells (HUVECs) were purchased from Cambrex (East Rutherford, NJ, USA). HUVECs were cultured as described previously (Park et al. 2016).

### Cell viability assay

HUVECs were cultured in 0.1% gelatin-coated plate at approximately 80% confluence. The cells were starved in M199 medium with 1% FBS for 6 h. Then, the cells were incubated with various doses of hydrangenol in the presence or absence of VEGF (20 ng/mL) for 24 h. The 3-(4,5-dimethylthiazol-2-yl)-2,5-diphenyltetrazolium bromide (MTT) assay was modified and used to determine the effect of hydrangenol on the cell viability of HUVECs.

### [^3^H] Thymidine incorporation

HUVECs were plated onto 0.1% gelatin-coated plates for 24 h, followed by starvation in M199 medium supplemented with 1% FBS. The cells were treated with indicated amounts of hydrangenol in the presence and absence of VEGF (20 ng/mL) for 24 h. Then, 1 Ci/mL of [*methyl*-^3^H]thymidine (New England Nuclear; Boston, MA) was added and incubated for 4 h. The incorporated [^3^H]thymidine was precipitated using cold 10 trichloroacetic acid, solubilized in 0.2 M NaOH, and counted using a scintillation counter (Perkin Elmer, Akron, OH, USA).

### Cell cycle analysis (FACS)

HUVECs were collected and fixed with 70% ethanol. The cells were fixed and stained with propidium iodide (PI) and subjected to cell cycle analysis as reported previously (Park et al. 2016). The phase distribution of the cell cycle was measured using FACStar flow cytometer (BD Biosciences, San Jose, CA) equipped with the BD Cell Fit software.

### Immunoblots, immunoprecipitation, and immune complex kinase assays

To investigate protein effectors associated hydragenol activity, western blotting, immunoprecipitation, and immune complex kinase assays were performed as described previously (Park et al. 2016).

### Endothelial cell colony tube formation assay

HUVECs were mixed for 12 h in growth factor-reduced Matrigel (Collaborative Biomedical Products, Bedford, MA, USA) in the presence or absence of hydrangenol in M199 medium containing 1% FBS and VEGF (20 ng/mL). Tubular structure formation was monitored by photography using a phase-contrast microscope. The tube length of HUVECs was measured using an Image-Pro Plus (Media Cybermetics, Silver Spring, MD, USA).

### Wound-healing migration, invasion, and zymographic assays

Cells (3×10^5^/well) were cultured in 6-well plates. To exclude the possibility of proliferation-mediated migration, cells were pre-incubated with 5 μg/mL of mitomycin C (Sigma-Aldrich) for 2 h. Thereafter, hydrangenol-mediated cellular migration and invasion were assessed and gelatinase activity was measured via zymography, as reported previously (Park et al. 2016). Morphological changes in cells after triacanthine treatment were confirmed via microscopy images acquired using an inverted microscope at 40× magnification.

### Aortic ring assay

The aortas were isolated from C57BL/6 mice (age, 2 months) and sectioned into 1–1.5-mm-long rings and then placed on Matrigel pre-coated wells. Each well containing the aortic rings was incubated in a medium containing VEGF (50 ng/mL) in the presence and absence of hydrangenol (10 mM). After 9 days, microvessel formation was measured using a standard light microscope that was equipped with Image-Pro Plus software (Media Cybernetics, Rockville, MD, USA). All animal experiments were performed with the approval of the Animal Care and Use Committee of Chungbuk National University.

### Statistical analysis

Values were presented as ±SEM obtained from experiments repeated at least thrice. Statistical analysis for two different groups was performed using unpaired Student's *t*-test. Analysis for more than two groups was performed using one-way analysis of variance test; Tukey's multiple comparison test was also performed subsequently. Statistical significance was considered at *p *< 0.05.

## Results

### Hydrangenol inhibits the VEGF-stimulated proliferation of HUVECs

**Figure 1.**
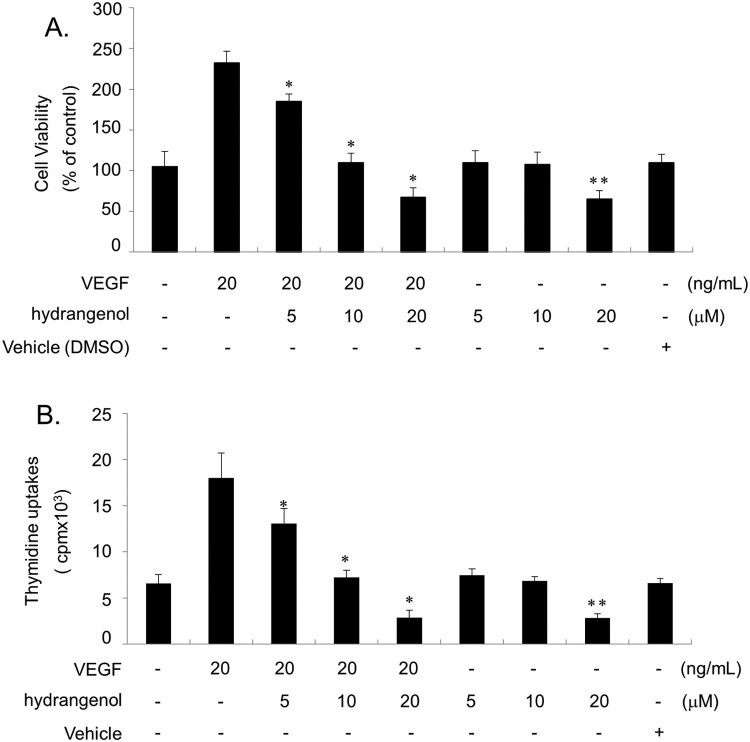
Inhibitory effect of hydrangenol on VEGF-stimulated HUVECs proliferation. After starvation for 6 h, HUVECs were incubated with hydrangenol (0, 5, 10, and 20 μM) for 40 min before stimulation with or without VEGF (20 ng/mL) for 24 h. (A) Cell viability was measured using the MTT assay. (B) Cell proliferation was measured using the thymidine incorporation assay. Values were presented as ±SEM from experiments repeated thrice. *p < 0.05, compared with VEGF treatment and **p < 0.01, compared with no treatment.

Because the proliferation of endothelial cells is the initial step toward angiogenesis, we investigated the effect of hydrangenol on HUVECs proliferation. Cells were incubated with different concentrations of hydrangenol (0, 5, 10, and 20 µM) in the presence or absence of VEGF (20 ng/mL). After 24 h, cell viability was quantified using MTT colorimetric assay. Treatment with VEGF effectively stimulated HUVECs proliferation up to 220% when compared with the control. However, the pro-proliferative effect of VEGF was significantly inhibited by the hydrangenol treatment in a dose-dependent manner. Owing to the treatment with 20 µM hydrangenol, VEGF-mediated HUVECs proliferation decreased approximately by 70% (Figure 1A). Treatment with hydrangenol alone indicated that hydrangenol concentration up to 10 µM had no effect on HUVECs proliferation; however, at 20 µM, HUVECs proliferation was reduced by approximately 40% as opposed to the control group (Figure 1A). Results from the [H^3^] thymidine incorporation assay were similar with those of the MTT assay, demonstrating a dose-dependent response to hydrangenol treatment (Figure 1B).

### Hydrangenol inhibits the transition of the G1 to S-phase of the cell cycle in HUVECs stimulated by VEGF

**Figure 2.**
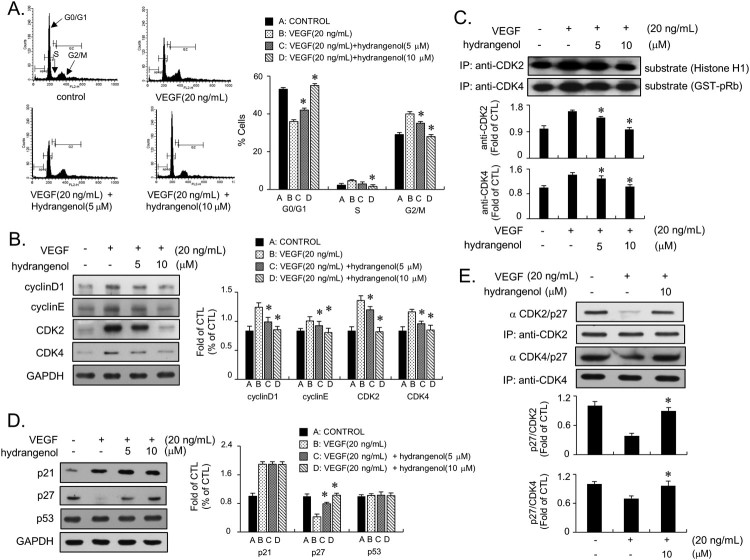
Hydrangenol induces G1-cell cycle phase arrest via p27KIP1 expression in VEGF-treated HUVECs. (A) Histograms from flow cytometry analysis of VEGF-stimulated HUVECs in the presence and absence of hydrangenol. The graph shows the distribution of cell populations in each cell cycle phase. (B) Immunoblots of cell cycle regulators in the G1-cell cycle phase. Hydrangenol-mediated changes in the levels of cyclin D1, cyclin E, CDK2, and CDK4 in VEGF-stimulated HUVECs were measured and presented in the bar graph. (C) Kinase assay was performed to evaluate the effect of hydrangenol on CDK2 and CDK4 activities. Cell lysates were collected and immunoprecipitated using anti-CDK2 and anti-CDK4 antibodies. Kinase activities of CDK2 and CDK4 were measured using histone H1 and GST-Rb as substrates, respectively. (D) Immunoblots for cell cycle inhibitors. Levels of p21WAF1, p27KIP1, and p53 were measured in VEGF-stimulated HUVECs treated with and without hydrangenol. The bar graphs were presented as a fold change compared with the control. (E) Cell lysates were immunoprecipitated using anti-CDK2 and anti-CDK4 antibodies, and then the immunoprecipitates were performed by the immunoblot assay using an anti-p27KIP1 antibody. All data are reported as means ± SE from three independently repeated experiments. *p < 0.05 compared with VEGF treatment.

To understand the mode of action of hydrangenol, we investigated the distribution of cell population in each phase of the cell cycle in response to hydrangenol treatment (0, 5, and 10 µM) in VEGF-stimulated HUVECs. As shown in Figure 2(A), histograms obtained from flow cytometry analysis indicated that hydrangenol disturbed the G1- to S-phase progression of cell cycle in HUVECs stimulated by VEGF in a concentration-dependent manner (Figure 2A). Treatment with hydrangenol led to the accumulation of cells in the G1-cell cycle phase, which causes a reduction in the population of cells at the G2/M-cell cycle phase (Figure 2A). This indicates that hydrangenol may act as a G1-cell cycle blocker in angiogenesis. Based on this result, we examined the key regulators in the G1-cell cycle phase including cyclin D1, cyclin E, CDK2, CDK4, p21WAF1, p27KIP1, and p53. HUVECs were incubated with hydrangenol at the indicated concentrations in the presence and absence of VEGF for 24 h, after which immunoblot analysis was performed. HUVECs incubated with VEGF significantly induced the expression of proteins, namely cyclin D1, cyclin E, CDK2, and CDK4; however, hydrangenol treatment reversed the VEGF-mediated increase in protein expression level to that associated with the control level (Figure 2B). To confirm this result, we examined kinase activities of CDKs in hydrangenol-treated HUVECs both in the presence and absence of VEGF. VEGF treatment promoted kinase activities of CDK2 and CDK4 in HUVECs (Figure 2C). Incubation with hydrangenol significantly suppressed the VEGF-mediated activation of both kinases (Figure 2C). Because various chemotherapeutic reagents promote the expression of cyclin-dependent kinase inhibitors, we subsequently examined whether hydrangenol affects cell cycle inhibitors including p21WAF1, p27KIP1, and p53. HUVECs stimulated with VEGF significantly upregulated the expression of p21WAF1 (Figure 2D). Upregulated p21WAF1 expression was not affected by hydrangenol treatment (Figure 2D). Meanwhile, p27KIP1 expression was significantly downregulated by VEGF; interestingly, hydrangenol treatment reversed the VEGF-mediated downregulation effect on p27KIP1 expression, such that it became equivalent to the expression in the control group (Figure 2D). The expression of p53 protein was unchanged by hydrangenol treatment (Figure 2D). To confirm this result, we examined the effect of hydrangenol on protein complex formation between p27KIP1 and CDKs using the IP assay. We pulled down the protein complex with CDK2 or CDK4, followed by immunoblots with the anti-p27KIP1 antibody. As shown in Figure 2(E), VEGF treatment significantly reduced the amount of immunoprecipitated p27KIP1; however, co-incubation with hydrangenol restored p27KIP1 expression level equal to that of the control group (Figure 2E). These results clearly demonstrate that hydrangenol inhibits the VEGF-mediated HUVECs proliferation by arresting the p27KIP1-dependent G1-cell cycle phase.

### Hydrangenol inhibits phosphorylation of ERK1/2, AKT, eNOS, and VEGFR-2 in VEGF-stimulated HUVECs

**Figure 3.**
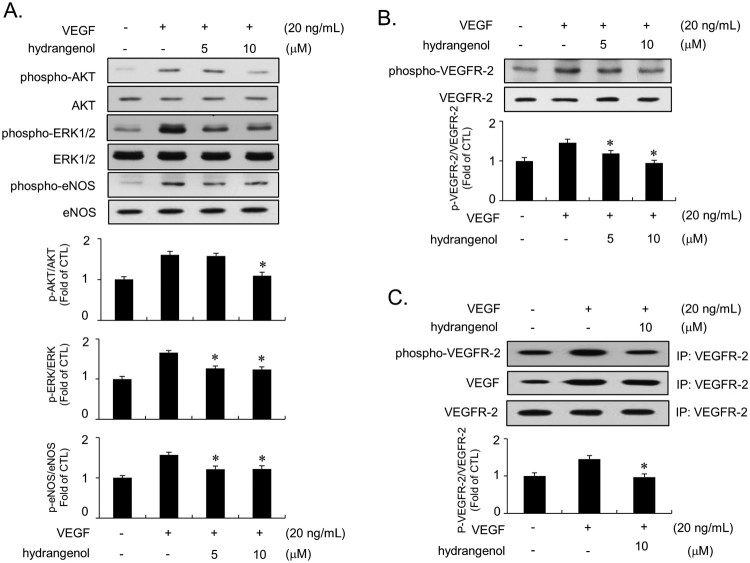
Inhibitory activity of hydrangenol against the phosphorylation of ERK1/2, AKT, eNOS, and VEGFR-2 in VEGF-stimulated HUVECs. HUVECs were pre-incubated with indicated concentrations of hydrangenol, followed by incubation in the presence of VEGF. (A) Immunoblots were performed using antibodies specific for phospho-ERK1/2, ERK1/2, phospho-AKT, AKT, phospho-eNOS, and eNOS. (B) Effect of hydrangenol on VEGFR-2 was assessed by the immunoblot assay, which employed anti-phospho VEGFR-2 antibody. (C) Immunoprecipitation was performed using the anti-VEGFR-2 antibody, and the immunoblot assay was subsequently performed using the phospho-VEGFR, VEGF, and total VEGFR-2 (input) antibodies. Values in bar graphs were presented as a fold change compared with the control group (untreated). Statistical analysis was performed using unpaired Student's *t*-test for VEGF alone vs. VEGF + hydrangenol-treated groups. Values were presented as ±SEM from three independently repeated experiments. Values at *p < 0.05 were considered significant.

Previously, early responsive regulators such as ERK1/2, AKT, or eNOS have been reported to participate in the angiogenic response of HUVECs stimulated by VEGF (Dimmeler et al. 1999). To investigate whether hydrangenol affects the early responsive regulators, we examined the phosphorylation of ERK1/2, AKT, and eNOS in VEGF-stimulated HUVECs both in the presence and absence of hydrangenol. Immunoblots demonstrated that VEGF promotes ERK1/2, AKT, and eNOS phosphorylation (Figure 3A). Hydrangenol treatment suppressed the VEGF-mediated phosphorylation of the effectors (Figure 3A). Subsequently, we investigated if hydrangenol inhibits VEGFR-2 phosphorylation because VEGF reportedly promotes angiogenic response by phosphorylating VEGFR-2 in endothelial cells (Lamalice et al. 2007). Hydrangenol treatment given to VEGF-stimulated HUVECs inhibited VEGFR-2 phosphorylation (Figure 3B). To validate this result, we performed IP assay using total VEGFR-2 and subsequently performed immunoblot assay using anti-phosphorylated VEGFR2, anti-VEGF, and anti-VEGFR-2 (input). As shown in Figure 3(C), hydrangenol markedly inhibited VEGFR-2 phosphorylation without affecting VEGF or total VEGFR-2 levels (Figure 3C). These results suggest that hydrangenol inhibits angiogenesis by hampering the VEGFR-2 phosphorylation in the cytoplasm of HUVECs.

### Hydrangenol inhibits colony tube formation, migration, and invasion in VEGF-stimulated HUVECs

**Figure 4.**
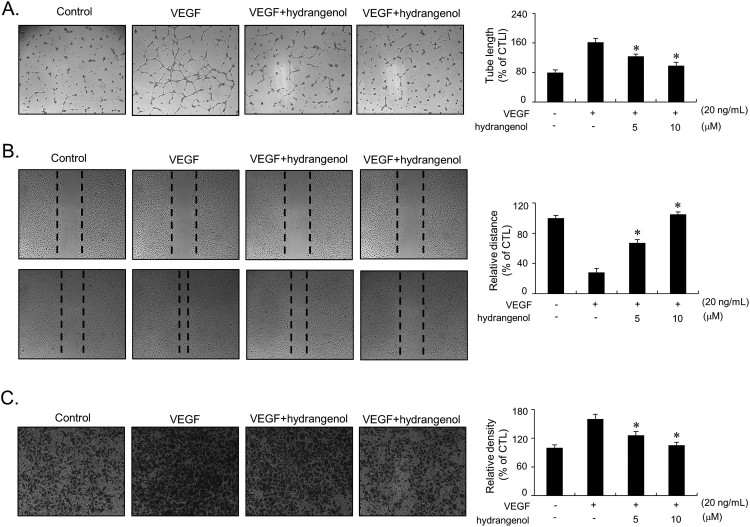
Inhibitory activity of hydrangenol against capillary-like tube formation, migration, and invasion in HUVECs stimulated by VEGF. (A) HUVECs grown on Matrigel-coated plates were treated with hydrangenol for 1 h, followed by VEGF treatment. Capillary-like tube structure was measured after 24 h using Image-Pro Plus. (B) HUVECs were incubated with hydrangenol (0, 5, and 10 μM). Cells were scratched and treated with VEGF (20 ng/mL) for 24 h. Cell morphology was photographed using an inverse light microscope. The relative migrated distance was measured and plotted on a graph and further compared with that of the control group (untreated). (C) Serum-starved HUVECs were plated on to the upper chamber of transwell plates. Cells were incubated with hydrangenol, followed by VEGF addition (20 ng/mL) and incubation for 24 h. Cells passing through the transwell membrane were visualized and quantified by staining with crystal violet. Values in the bar graphs were presented as a fold change in comparison with those of the control group (untreated). Statistical analysis was performed using unpaired Student's *t*-test for VEGF alone vs. VEGF + hydrangenol-treated groups. Values were presented as ±SEM of the three independently repeated experiments. Values at *p < 0.05 were considered significant.

Upon stimulation by pro-angiogenic factors, endothelial cells undergo morphological changes and form capillary-like tubular structures (Bussolino et al. 1997). We examined whether hydrangenol inhibits the tubular structure formation induced by VEGF in HUVECs. VEGF treatment promptly induced tube formation (Figure 4A). Co-incubation with hydrangenol significantly inhibited the VEGF-mediated tube formation (Figure 4A). Tube length measurement also revealed that hydrangenol effectively inhibited the VEGF-mediated tubular structure formation in a dose-dependent manner (Figure 4A). We then investigated whether hydrangenol inhibits the migratory and invasive potential of VEGF-stimulated HUVECs because the migration and invasion of endothelial cells are critical steps in new blood vessel formation process (Lamalice et al. 2007). Cells were grown to 80–90% confluence, and wound-healing assay was performed in culture medium with or without VEGF in the presence and absence of hydrangenol. As shown in Figure 4(B), the migration of HUVECs was significantly promoted by the treatment of VEGF, whereas co-incubation with hydrangenol effectively inhibited the VEGF-mediated migratory potential of HUVECs in a dose-dependent manner. Using Martigel-coated transwell chambers, we also examined the effect of hydrangenol on the invasive potential of VEGF-stimulated HUVECs. VEGF treatment accelerated HUVECs penetration through the transwell membrane (Figure 4C). Hydrangenol significantly inhibited the VEGF-mediated invasion of HUVECs in a dose-dependent manner (Figure 4C).

### Hydrangenol inhibits VEGF-stimulated MMP-2 activity in HUVECs and suppresses VEGF-induced microvessel sprouting in *ex vivo* aortic ring assay

**Figure 5.**
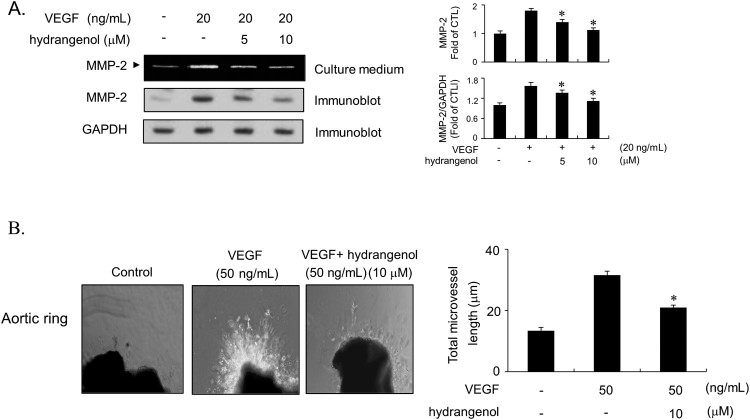
Hydrangenol inhibits VEGF-stimulated MMP-2 activity in HUVECs *in vitro*, and VEGF induced the sprouting of microvessels in aortic rings *ex vivo*. (A) Serum-starved HUVECs were grown in a conditioned medium in the presence and absence of hydrangenol for 40 min, followed by the VEGF treatment. The conditioned culture medium was collected and subjected to zymography (upper panel). Immunoblot assay was performed to measure MMP-2 protein expression (middle panel). GAPDH was used as a loading control (bottom panel). (B) Aortic vessel segments from mice were embedded in Matrigel-coated plates, followed by the treatment with or without hydrangenol both in the presence and absence of VEGF (50 ng/mL) for 9 days. The total length of microvessels was measured and quantified using Image-Pro Plus software (Media Cybernetics, USA). Statistical analysis was performed using unpaired Student's *t*-test for VEGF alone vs. VEGF + hydrangenol-treated groups. Values were presented as ±SEM of three independently repeated experiments. Values at *p < 0.05 were considered significant.

During the angiogenic process, endothelial cells respond to the pro-angiogenic stimuli by secreting matrix metalloproteases (MMPs), particularly MMP-2, that degrade ECM, and thus migrate and invade the basement membrane, which causes new blood vessel formation (Bussolino et al. 1997). Therefore, we investigated the change in MMP-2 activity in VEGF-induced HUVECs in response to hydrangenol treatment using a gelatin zymography assay. As shown in Figure 5(A), the treatment of HUVECs with VEGF markedly promoted MMP-2 activity. The incubation of cells with hydrangenol significantly and dose-dependently inhibited the VEGF-mediated activation of MMP-2 (Figure 5A). Immunoblots for MMP-2 also indicated that hydrangenol effectively inhibited the expression of MMP-2 protein (Figure 5A). Based on these results, we investigated the antiangiogenic activity of hydrangenol using the *ex vivo* aortic ring assay. To this end, we measured sprouting capacity from the aortic rings by determining the total length of microvessels. VEGF treatment significantly promoted the sprouting of microvessels from aortic rings (Figure 5B). In contrast, hydrangenol treatment effectively inhibited VEGF-mediated microvessel formation (Figure 5B). Taken together, these results clearly indicate that hydrangenol efficiently suppresses VEGF-stimulated angiogenesis both *in vitro* and *ex vivo*.

## Discussion

Metastasis of cancer cells is closely associated with angiogenesis stimulated by angiogenic activators such as PDGF-BB, TGF-β1, FGF, HGF, NOS, COX-2, IL-5, and VEGF (Carmeliet & Jain 2000). Among these factors, VEGF has been well characterized as a key regulator of angiogenesis and thus, well accepted as a critical mitogen for the proliferation of vascular endothelial cells both *in vitro* and *in vivo* (Yancopoulos et al. 2000). Therefore, small molecules targeting angiogenesis could be a good strategy to manage angiogenesis-mediated diseases.

Hydrangenol is one of the bioactive compounds obtained from plants belonging to the Hydrangeaceae family. Recently, hydrangenol has been intensively studied due to its bioactivities against various diseases including liver diseases, diabetes, and malaria (Kamei et al. 2000). Despite intensive studies on hydrangenol, researches on the effect of hydrangenol against cancers are very limited. Previously, we have reported that hydrangenol has potent anticancer activity against bladder cancer, which is the fourth most common cancer in the United States of America (Shin et al. 2018). As a follow-up study, we investigated the potential antiangiogenic activity of hydrangenol using both *in vitro* HUVECs and *ex vivo* aortic ring systems stimulated by VEGF as models of angiogenesis.

We demonstrated that hydrangenol has an antiproliferative activity against HUVECs stimulated by VEGF, a potent mitogen that promotes angiogenesis. MTT and thymidine uptake assays suggested that the inhibitory activity of hydrangenol is concentration dependent up to 20 M. Interestingly, HUVECs treated with 20 M hydrangenol alone also inhibited cell proliferation by approximately 40% as opposed to the control, suggesting cytotoxic function at >20 M. Therefore, we established EC_50_ at 10 M. The flow cytometry analysis of VEGF-stimulated HUVECs revealed that VEGF promotes cell cycle progression, thereby increasing the prevalence of cells in the S- and G2/M-cell cycle phases. However, hydrangenol treatment of cells led to an increase in cells present in the G1-cell cycle phase and a reduction in cells present in the S- and G2/M phases of the cell cycle. This data was interesting because many FDA-approved cancer drugs including paclitaxel and tamoxifen are G1-cell cycle blockers (Lin et al. 1998) as well as angiogenesis inhibitors (Bocci et al. 2013). Among regulators of the G1-cell cycle phase, cyclin D1, cyclin E, CDK2 and CDK4, were significantly inhibited by hydrangenol in VEGF-induced HUVECs. Measurement of the kinase activity of both CDK2 and CDK4 suggested that hydrangenol not only inhibited protein expression but also kinase activity. The protein expression of p21WAF1, both positive and negative cell cycle regulators, was upregulated by the stimulation of VEGF; however, hydrangenol addition did not affect protein expression level. The protein level of p53 remained unchanged regardless of hydrangenol treatment. In contrast, p27KIP1 protein level was significantly upregulated by hydrangenol treatment in the presence of VEGF as opposed to that during VEGF treatment alone, suggesting that the regulation of G1-cell cycle phase by hydrangenol was a p27KIP1-dependent process, but not p21WAF1- or p53-dependent. Results from the IP assay performed using anti-CDK2 and CDK4 along with those from the immunoblot assay performed using p27KIP1 also supported the notion that hydrangenol-mediated accumulation in the G1-cell cycle phase is greatly associated with p27KIP1 expression. VEGF activates VEGFR-2, which in turn activates early responsive regulators including ERK1/2, AKT, and eNOS in HUVECs. Hydrangenol treatment suppressed the VEGF-mediated transduction of VEGF/VEGFR-2/ERK1/2/AKT/eNOS cascades. IP analysis revealed that the amount of VEGF ligand bound onto the VEGFR-2 was unchanged after hydrangenol treatment. Given that hydrangenol inhibits the downstream signaling regulators of VEGFR-2 without inhibiting the binding capacity of VEGF, our data demonstrate that hydrangenol may inhibit VEGFR-2 via an allosteric regulatory mechanism. Taken together, these results suggest that hydrangenol inhibits angiogenesis by suppressing ERK1/2, AKT, and eNOS activities and by directly blocking VEGFR-2 activation.

During angiogenesis, endothelial cells secrete proteolytic enzymes to degrade the basement lamina by stimulating angiogenic factors. These cells proliferate to form a three-dimensional capillary-like tubular structure and shape new blood vessels (Lamalice et al. 2007). In our study, hydrangenol exhibited potent inhibitory activity against VEGF-mediated migration, invasion, and capillary-like tube formation in HUVECs. Because previous reports have demonstrated that VEGF only activates MMP-2 and not MMP-9 in HUVECs (Genersch et al. 2000), we next focused on the effect of hydrangenol on MMP-2 activity in VEGF-treated HUVECs. Gelatin zymography and immunoblot analysis revealed that hydrangenol significantly inhibited both VEGF-induced expression and -enzymatic activity of MMP-2 in HUVECs. Taken together, our results show that the inhibitory activity of hydrangenol against migration, invasion, and capillary-like tube formation in VEGF-induced HUVECs is partially owing to the inhibition of MMP-2 activity. Based on the *in vitro* results, we evaluated the antiangiogenic activity of hydrangenol using excised vascular aortic rings from mice. VEGF effectively induced the sprouting of microvessels from aortic rings after 9 days of *ex vivo* transplantation. Hydrangenol treatment inhibited the formation of microvessel outgrowth, suggesting that hydrangenol has potent inhibitory activity against angiogenesis both *in vitro* and *ex vivo*.

In conclusion, these data provide insights that hydrangenol impeded VEGF-induced HUVECs proliferation by arresting the p27KIP1-dependent G1-cell cycle phase. Hydrangenol inhibited VEGFR2 phosphorylation and thereby blocked ERK1/2/AKT/eNOS angiogenic signaling pathways in VEGF-treated HUVECs. Additionally, hydrangenol repressed capillary-like tube formation, migration, and invasion in HUVECs induced by VEGF via suppression of MMP-2 activity. Furthermore, VEGF-stimulated angiogenic potential was proved by *ex vivo* aortic ring assay system. Thus the present results demonstrate that hydrangenol may be a potential preventive agent for angiogenesis-related diseases including cancer and vascular disorders.
